# Out of Plane Distortions of the Heme *b* of *Escherichia coli* Succinate Dehydrogenase

**DOI:** 10.1371/journal.pone.0032641

**Published:** 2012-02-29

**Authors:** Quang M. Tran, Carmen Fong, Richard A. Rothery, Elena Maklashina, Gary Cecchini, Joel H. Weiner

**Affiliations:** 1 Membrane Protein Research Group, Department of Biochemistry, University of Alberta, Edmonton, Alberta, Canada; 2 Molecular Biology Division, Veterans Affairs Medical Center, San Francisco, California, United States of America; 3 Department of Biochemistry and Biophysics, University of California San Francisco, San Francisco, California, United States of America; Université Joseph Fourier, France

## Abstract

The role of the heme *b* in *Escherichia coli* succinate dehydrogenase is highly ambiguous and its role in catalysis is questionable. To examine whether heme reduction is an essential step of the catalytic mechanism, we generated a series of site-directed mutations around the heme binding pocket, creating a library of variants with a stepwise decrease in the midpoint potential of the heme from the wild-type value of +20 mV down to −80 mV. This difference in midpoint potential is enough to alter the reactivity of the heme towards succinate and thus its redox state under turnover conditions. Our results show both the steady state succinate oxidase and fumarate reductase catalytic activity of the enzyme are not a function of the redox potential of the heme. As well, lower heme potential did not cause an increase in the rate of superoxide production both *in vitro* and *in vivo*. The electron paramagnetic resonance (EPR) spectrum of the heme in the wild-type enzyme is a combination of two distinct signals. We link EPR spectra to structure, showing that one of the signals likely arises from an out-of-plane distortion of the heme, a saddled conformation, while the second signal originates from a more planar orientation of the porphyrin ring.

## Introduction

During aerobic metabolism, *Escherichia coli* succinate dehydrogenase (SdhCDAB, complex II) catalyzes the oxidation of succinate to fumarate and ultimately transfers the chemical energy to the ubiquinone pool to be used in the terminal reduction of oxygen by cytochrome oxidase. Located in the bacterial inner membrane, the enzyme consists of two domains: a soluble SdhAB dimer that is bound to the inner membrane by the SdhCD transmembrane domain. The oxidation of succinate occurs at a flavin adenine dinucleotide (FAD) cofactor that is covalently attached to a histidine in the SdhA subunit. The oxidation of succinate to fumarate releases two electrons (and 2 H^+^) that are transferred directly to the FAD cofactor, itself a two-electron (and hydrogen) carrier. These electrons must sequentially tunnel through a series of three iron-sulfur ([Fe-S]) clusters – a [2Fe-2S] FS1, [4Fe-4S] FS2, and [3Fe-4S] FS3, in that order – that form an electron transfer relay through the SdhB subunit. We have previously shown that electrons from FS3 can directly reduce ubiquinone at a quinone-binding site (Q-site) located 8.4 Å (edge-to-edge distance) from FS3 [1]. A complete 2-electron reduction of the quinone proceeds via a stable, EPR-detectable ubisemiquinone intermediate [1].

Located in the middle of the membrane, sandwiched between the SdhC and SdhD subunits is a bis-histidine-coordinated *b*-type heme. One histidine residue from each of the SdhC and SdhD subunits provides the two ligands to the heme iron. The heme is conserved in the complex II family of enzymes [2]–[5] but it does not lie in the direct pathway of the electron transfer relay between the FAD and Q-site. While the heme cycles through redox states in the presence of succinate and quinones, this redox activity does not appear to be essential for enzyme function as a heme-free variant retains a significant amount of catalytic activity and is capable of supporting the growth of *E. coli* on succinate [6]. The *E. coli* Sdh paralogue fumarate reductase (FrdABCD, Frd) lacks heme and catalyzes the reduction of fumarate to succinate. However, over-expressed FrdABCD can catalyze succinate∶ubiquinone oxidoreductase activity sufficient to complement an *sdh* deletion strain [7]. Although it is clear that the heme is necessary to stabilize the structure of the isolated Sdh enzyme [6], [8] the exact function of this heme cofactor in electron transfer remains to be determined.

The mitochondrial respiratory chain is a significant source of free radicals but the contribution of complex II to the overall generation of reactive oxygen species (ROS) is relatively minor. Through an auto-oxidative process, *E. coli* Sdh normally produces low quantities of ROS in the form of superoxide [9], however, mutations around the Q-site in *E. coli* (and also *Saccharomyces cerevisiae* Sdh) can lead to an increased rate of superoxide production [10], [11]. It has been suggested that the heme is behaving as an electron buffer to prevent the production of ROS from a Q-site-stabilized ubisemiquinone [4] but experimental evidence for this is lacking.

The redox potential (E*_m,7_*) of the heme in *E. coli* Sdh is +20 mV [12]. This is in contrast to published values for the heme in bovine complex II which has an *E*_m,7_ of −185 mV [13]. It is for this reason that the heme in mammalian complex II cannot be reduced in the presence of succinate [13] but the heme of *E. coli* Sdh can be reduced by succinate and oxidized by ubiquinone [1], [14].

The loss of the heme in *E. coli* Sdh is accompanied by a decrease in catalytic rates [6], which may arise from improper, yet competent, folding of the membrane subunits, but could also result from dysfunctional electron tunneling pathways. To examine the latter, we used a directed mutagenesis approach to alter the midpoint potential of the heme and measured the effects of altered heme redox chemistry on enzyme activity and ROS production. A biophysical characterization of these Sdh variants reveals the heme can exist in two distinct conformations that differ in planarity of the porphyrin ring. This is the first evidence of structural heterogeneity of the heme in this family of enzymes and the significance of out-of-plane distortion of the porphyrin ring is discussed.

## Materials and Methods

### Site-directed mutagenesis

The EcoRI/BamHI fragment of the pFAS plasmid [7] containing the *sdhCDAB* operon was subcloned into the cloning vector pTZ18R ((Amp^R^, *lacZ′*), GE healthcare). This plasmid was used as a template for site-directed mutagenesis to introduce the required single mutations according to the QuikChange procedure (Stratagene). The mutagenic oligonucleotides (Integrated DNA Technologies) used in the PCR reactions are listed in **Table S1**. All mutations were confirmed by DNA sequencing (DNA Core Facility, Department of Biochemistry, University of Alberta). In order to create double mutations, the KpnI/BamHI fragment of the plasmid bearing the SdhC^V87D^ mutation was cut out and reinserted into the plasmid containing the appropriate second mutation. Once all mutagenesis was complete, the EcoRI/SphI fragment was subcloned back into pFAS and transformed into *E. coli* strain DW35 (Δ*frdABCD*, *sdhC*::*kan*), a strain that does not contain any wild type Sdh or Frd [15]. All genetic manipulations were done in the *E. coli* strain TG1 ((*hsd*Δ5 *thi* Δ(*lac-proAB*) *F*′[*traD*36 *proAB*+*lacI*q *lacZ*ΔM15]), Amersham Biosciences)).

### Isolation of crude membranes

*E. coli* DW35 expressing Sdh variants was grown in TB media overnight for 16–18 hours using a 1∶100 starter inoculum as reported previously [1]. After harvesting, cells were lysed by multiple passes through an Avestin Emulsiflex in the presence of 20 µM phenylmethylsulfonyl fluoride. The bacterial inner membrane fraction was isolated using differential centrifugation as previously described [1]. During the procedure, membranes were incubated at 30°C for 15 minutes with 1 mM malonate to remove oxaloacetate bound at the FAD site of Sdh. All cells and membrane isolations were maintained in 100 mM MOPS/5 mM EDTA buffers.

### Protein quantitation

To assess the overall expression and assembly of SdhCDAB, all membrane samples were examined after electrophoresis on a 12.5% SDS-PAGE gel [6]. 45 µg of membranes were loaded alongside a low molecular weight ladder (Bio-Rad) and stained with Coomassie blue. Protein concentrations were quantitated using the Lowry assay [16] and Sdh levels quantitated by measuring covalent-flavin [17].

### Enzyme activity assays

The succinate/Q_0_ and plumbagin/fumarate assays were performed as described previously [1]. In order to calculate k_cat_ and K_m_ values, the Q_0_ or plumbagin concentration was varied from 0.07 mM to 0.3 mM and data was plotted to an Eadie-Hofstee graph. The succinate/phenazine methosulfate (PMS)/3-(4,5-dimethylthiazol-2-yl)-2,5-diphenyltetrazolium bromide (MTT) assay was performed as before [14].

### UV-VIS and EPR spectroscopy

Reduced *minus* oxidized absorbance spectra were recorded on a Hewlett Packard 8453 UV-VIS diode array spectrophotometer. Membranes were diluted to a protein concentration of 0.5 mg mL^−1^ in degassed 100 mM MOPS/5 mM EDTA buffer (pH 7.0). Samples were reduced with either excess sodium dithionite, or 9 mM sodium succinate.

Potentiometric titrations were performed as previously described [1]. Protein concentrations within the EPR samples varied from 20–30 mg mL^−1^. Oxidized membrane samples were made by adding 2,6-dichlorophenolindophenol (DCPIP) to a final concentration of 0.8 mM [6]. All samples were made using standard 3 mm diameter quartz EPR tubes and stored in liquid nitrogen until anazlyzed. EPR spectroscopy was performed on either a Bruker Elexsys E500 or Bruker ESP300E EPR Spectrophotometer, each equipped with an Oxford Instruments ESR900 flowing helium cryostat. Both [3Fe-4S] and heme *b* EPR spectra were recorded at 12 K, 20 mW at 9.47 GHz, 10G_pp_.

### Measurement of reactive oxygen species

A cytochrome *c* reduction assay was used to quantitate the production of superoxide during enzyme turnover [9]. A disc assay was used to measure the susceptibility of *E. coli* to oxidative stress. Sloppy agar overlays of the appropriate *E. coli* culture were made. 0.7 cm-wide discs of Whatman no. 1 filter paper were placed atop the overlays and 3 µl of a 0.5 mM or 0.25 mM solution of methyl viologen was added to the centre of each disc. The plates were incubated overnight at 37°C and the diameter of the resulting zone of inhibition was measured along two orthogonal axes.

## Results and Discussion

### Selection of Residues to Mutate

**Figure 1:**
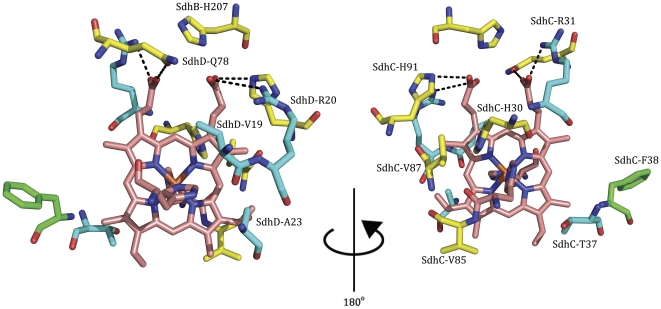
Residues selected for mutation. Upon mutation, residues that result in a shift towards the “sharp” EPR spectrum are colored in yellow while positions causing a shift towards the “broad” EPR spectrum are colored in blue. Residues that did not affect the EPR spectrum when mutated are shown in green. (pdb: 1NEK [4]).

Using site-directed mutagenesis, we modulated the redox potential of the heme by replacing residues within its second coordination sphere. We selected non-conserved residues in the immediate vicinity of the heme (within 5 Å of the heme edge) using the X-ray structure (pdb code: 1NEK [4]) as a guide, and focused on residues with sidechains pointed directly at the heme (**Figure 1**). When measuring effects on catalytic activity, we concentrated only on these mutations to non-conserved amino acids located away from the propionates and the Q-site, in order to avoid effects unrelated to the heme potential (**Table 1**). Another set of mutations was generated at residues positioned around the heme propionates; however, these variants were only characterized for their biophysical properties and were not included in our analysis of catalytic turnover. These latter variants are discussed below.

**Table 1:** Redox characteristics, physiological and non-physiological activity, and ROS assays of wild-type and Sdh variants.

Variant	E_m,7_ heme *b*	E_m,7_ [3Fe-4S]	k_cat_ Q_0_ (s^−1^)	k_cat_/K_m_ Q_0_ (×10^6^ M^−1^ s^−1^)	k_cat_ PMS-MTT(s^−1^)	k_cat_ PB (s^−1^)	Disc Assays (cm^2^)	ROS production (s^−1^)
SdhCDAB	20	60	42.9±0.4	0.29±0.06	19.9±0.2	39.6±1.5	1.73	2.0±0.4
D-V19D	2	55	30.0±3.3	0.23±0.03	23.2±0.8	n.d.	n.d.	n.d.
D-A23D	−5	40	35.7±1.3	0.26±0.01	20.9±0.7	n.d.	n.d.	n.d.
C-F38D	−10	50	38.1±2.6	0.28±0.04	20.9±0.6	35.7±1.1	1.19	1.2±0.4
C-V85D	−13	55	14.8±0.4	0.29±0.05	11.4±0.4	n.d.	n.d.	n.d.
C-T37I	−22	50	30.3±2.2	0.22±0.02	16.5±0.7	29.2±1.5	n.d.	n.d.
C-F38S	−25	40	37.3±4.7	0.24±0.06	20.4±0.8	n.d.	1.31	1.6±0.1
C-H30A/C-V87D	−30	45	29.7±3.1	0.17±0.03	21.2±0.4	n.d.	n.d.	n.d.
D-A23S	−40	60	27.1±2.0	0.16±0.02	11.0±0.3	27.9±1.1	n.d.	n.d.
C-V87D	−43	35	34.1±1.8	0.20±0.03	22.9±0.8	n.d.	n.d.	n.d.
C-V85T	−45	60	44.8±1.0	0.36±0.02	26.2±1.5	54.2±1.8	n.d.	2.8±0.3
C-H30G	−50	45	27.9±2.0	0.22±0.03	12.9±0.2	n.d.	1.17	0.8±0.1
C-H30A	−54	53	32.4±3.3	0.21±0.03	17.6±0.4	29.8±1.2	n.d.	n.d.
C-H30S	−62	40	35.3±2.0	0.21±0.04	21.5±0.9	n.d.	n.d.	n.d.
C-F38D/C-V87D	−80	60	13.3±1.7	0.05±0.01	15.9±0.2	21.6±1.0	1.61	0.8±0.1

Turnover numbers have all been normalized to FAD content. Activity data were averaged from at least three independent measurements.

In contrast to the soluble domains of *E. coli* Sdh and eukaryotic complex II, the membrane domains share very low sequence identity [2]. When conserved residues are mapped onto the available Sdh crystal structures, the majority of these residues in SdhC and SdhD are confined to key areas of the enzyme near the Q-site and heme propionates (not shown). Despite the fact that both share the same core 4-helix bundle-type structure, the individual residues along those helices show significant variation. However, the locations in three-dimensional space of the backbone α-carbons are well conserved even though the individual residues are not. Due to the bis-histidine coordination of the heme and common architecture conserved in both *E. coli* and eukaryotic enzymes, the amino acids side chains adjacent to the heme align well, spatially, when comparing the structures of *E. coli* Sdh to avian or porcine complex II (pdb codes 1ZOY [2] and 2H88 [3], respectively). We noticed that the heme binding pocket is much more polar in mitochondrial complex II than in *E. coli* Sdh. Of the residues we chose to mutate, many of the nonpolar residues were mutated to the polar residue found at the equivalent position in the mitochondrial enzyme.

### Expression and Membrane Assembly of Variant Sdh Enzymes

The mutations introduced into the SdhCD membrane domain did not have a detrimental effect on the expression and assembly of the enzyme as determined by SDS-PAGE analysis of crude membranes prepared from bacterial cultures over-expressing each of the variants (**Figure S1**). A standard covalent-flavin assay was used to quantify the amount of SdhA in each sample; levels of FAD varied between 1.5–2.5 nmol FAD mg^−1^ total protein and correlated well with SdhA levels seen on the SDS-PAGE gel.

We utilized the PMS-MTT assay to measure non-physiological succinate oxidation activity of the enzyme. This assay requires only a functional SdhAB dimer and thus it is a measure of overall expression and insertion into the membrane. As shown in **Table 1**, the PMS-MTT activity of the variants was similar to that of wild-type Sdh, although the SdhD^A23S^, SdhC^V87D^, and SdhC^H30G^ variants had somewhat lower activity. Thus, mutations in the membrane domain did not significantly alter the ability of the enzyme to oxidize succinate to fumarate.

### Determination of the Midpoint Potential of Heme *b* in Variant Sdh Enzymes

We used a combination of redox potentiometry and EPR spectroscopy to determine the midpoint potential of the heme in all Sdh variants (**Table 1**). We also measured the midpoint potential of the [3Fe-4S] FS3 cluster, which should be unaffected by mutations in the transmembrane domain. The midpoint potential of the FS3 cluster was not significantly altered in any of the Sdh variants (E*_m,7_* = +60 mV).

In wild-type *E. coli* Sdh, the midpoint potential of the heme is +20 mV. Using site-directed mutagenesis, we successfully generated variants with a wide range of heme potentials, from +2 mV down to −80 mV. In all the variants, the heme remained hexa-coordinate and low-spin. In some cases, a single amino acid residue difference was enough to drop the redox potential of the heme by as much as 75 mV and a combination of the SdhC^F38D^ and SdhC^V87D^ mutations had a cumulative effect, resulting in a 100 mV change.

As expected, the introduction of an Asp residue into the low dielectric environment of the heme resulted in drops of 20–60 mV in the heme potential depending on the position of the mutation. Negatively charged amino acids are expected to stabilize the oxidized form of the heme, which is cationic. This is consistent with results in synthetic heme protein maquettes in which the addition or subtraction of a single charged amino acid in the vicinity of a bis-His coordinated heme yielded a change in heme redox chemistry of approximately ±50 mV [18].

Interestingly, none of the mutations resulted in an increase in heme potential, even when the heme microenvironment is made more hydrophobic (as in the SdhC^T37I^ or SdhC^H30A^ variants). In these cases, the lower dielectric within the protein milieu might be expected to raise the *E*_m_ of the heme through stabilization of the electronically neutral ferrous heme state. Instead, the redox potential of the heme dropped 40–75 mV in these variants. This highlights the inherent unpredictability in these types of experiments. In these cases, the counterintuitive result may be due to increased solvent exposure of the heme (in the X-ray structures, SdhC^H30^ appears to shield the heme cofactor from nearby water molecules) or differential packing against the hydrophobic core, which may instead stabilize the oxidized form of the heme [18].

As noted above, a comparison of residues immediately next to the heme cofactor indicate that the heme binding pocket in the eukaryotic complex II is much more polar than in *E. coli* Sdh. In our experiments, individual amino acid mutations in *E. coli* Sdh can decrease the redox potential of the heme by up to 75 mV. Given the cumulative nature of these amino acid replacements, the increased dielectric environment is likely a major cause of the 200 mV difference between the midpoint potential of the heme in mitochondrial complex II (*E*_m,7_ = −185 mV [13]) and *E. coli* Sdh (+20 mV).

The low redox potential of the heme in mitochondrial complex II is very similar to that measured in the Sdh of *Paracoccus denitrificans* (*E*_m,7_ = −176 mV ([19]), a bacterium thought to be the possible ancient ancestor of the mitochondrion [20]. This range of heme potentials is not much higher than the potential of bis-imidazole-ligated heme *b* in solution (E*_m8.5_* = −235 mV) [21]. Considering the unusually high midpoint potential of the heme in *E. coli* Sdh and the ease by which mutations around it are able to lower its potential, it may be a case that in the *E. coli* enzyme, the heme-binding pocket has evolved to maximize the *E*_m_ of the heme. This suggests a non-structural role for the heme since its redox potential would be irrelevant in an exclusive role as a molecular chaperone. Since the heme in mitochondrial complex II is not reducible by succinate [13], but it can be succinate-reduced in *E. coli* Sdh [1], this suggests that if the heme in *E. coli* Sdh serves a non-structural role, this function is unlikely to be conserved in the mitochondrial enzyme.

### Visible Spectroscopy of Heme *b* in Sdh Variants

Each of our variants exhibit typical room temperature heme absorption spectra with Soret maxima at 410 nm and 427 nm in the oxidized and reduced state, respectively (data not shown). Normal β- and α- band absorbance maxima at 542 nm and 560 nm were also observed. This suggests that the heme environment is not dramatically altered as a result of the mutations. When succinate instead of dithionite was used to reduce the heme, the levels of reduced heme reflected the EPR-determined midpoint potential. In the SdhC^F38D^/SdhC^V87D^ double variant with the lowest redox potential, succinate-reducible heme comprises only 10% of wild-type Sdh level. This 10% signal may not derive entirely from reduced Sdh heme, and may be a result of redox equilibrium with the downstream hemes of the *E. coli* cytochrome oxidases [22] (**Figure S2**).

### Catalytic Characterization of Variant Enzymes

In order to assess the effect of heme potential on catalytic turnover, we measured the ability of the variant enzymes to reduce the ubiquinone analogue, Q_0_, in the presence of excess succinate. As shown in **Table 1**, all of the variants were catalytically active. Wild-type Sdh reduces Q_0_ with a k_cat_ of 43 s^−1^. Although most of the variant proteins had a decreased capacity to reduce quinone, we could not see a correlation between the midpoint potential of the heme and Q_0_-reductase activity, confirming that heme reduction is not essential for catalytic function. We only observed dramatic decreases in quinone reduction activity in the cases of the SdhC^V85D^ and SdhC^F38D^/SdhC^V87D^ variants, which may be caused by secondary effects to enzyme structure. In most cases, we measured a 10–40% decrease in the catalytic activity of the enzyme. This range of activity is reasonable since a complete loss of the heme cofactor only attenuates activity by roughly 50% [6]. The SdhC^V85T^ variant, which has a heme *E*_m,7_ of −45 mV (65 mV lower than wild-type Sdh), retained wild-type levels of Q_0_-reductase activity. As well, none of the mutations had a significant effect on the K_m_ for Q_0_. This suggests that quinone binding is not affected by any of the mutations, as predicted from their substantial distance from the Q-site. Additionally, there was no strict correlation between the catalytic efficiency of the quinone reduction (k_cat_/K_m_) and the midpoint potential of the heme (**Table 1**).

### Fumarate Reductase Activity of Variant Sdh Enzymes

SdhCDAB is able to catalyze the reverse reaction, the quinol-dependent reduction of fumarate, when supplied with a source of reducing equivalents. We measured the ability of the variant enzymes to catalyze the reduction of fumarate using the menaquinone analogue, reduced plumbagin, and as shown for the forward reaction, little correlation was observed between *E*_m_ and activity (**Table 1**). In all variant enzymes that we tested, plumbagin oxidation rates were directly proportional to Q_0_ reduction rates. Thus, we have shown that *in vitro*, modulating the midpoint potential of the *E. coli* Sdh heme does not have a definite effect on the ability of the enzyme to reduce quinone or oxidize quinol substrates.

### Reactive Oxygen Production *in vitro*

It has been suggested that the FAD is the major site of superoxide production in Sdh [9]. The high potential of the heme may allow it to draw electrons away from the FAD. Lowering the potential of the heme would increase ROS production as electrons accumulate at the FAD. Alternatively, the heme may act as an electron sink in order to prevent the formation of ROS via the stable ubisemiquinone intermediate. In this case, a reduced capacity for electrons at the heme should increase the lifetime of the ubisemiquinone intermediate and thus increase the production of superoxide from the Q-site.

We examined the effect of lower heme midpoint potential on the production of ROS by measuring the production of superoxide during enzyme turnover using a cytochrome *c* reduction assay (**Table 1**). Wild-type Sdh produces superoxide at a rate of approximately 2 s^−1^. All of the variant enzymes tested produced superoxide at a rate equivalent to wild-type Sdh when normalized to catalytic turnover, which was approximately 5% the rate of Q_0_ reduction. Thus, lowering the redox potential of the heme has no appreciable effect on the production of ROS in this enzyme.

### Reactive Oxygen Production *in vivo*

We determined whether these variant enzymes could be producing increased levels of superoxide *in vivo*. Disc assays were performed on DW35 cells expressing wild-type as well as the Sdh variants (**Table 1**). When 1.5 µmol of paraquat was added to the 0.6 cm filter discs, the zone of growth inhibition around the cells expressing wild-type Sdh was 1.7 cm^2^. The corresponding zones of inhibition surrounding the DW35 cells expressing the variant Sdh enzymes were in every case smaller when compared to those cells expressing wild-type Sdh. Paraquat sensitivity was not enhanced in any of the variant enzymes tested and similar results were obtained when 0.75 µmol of paraquat was used (not shown). These results mirror the *in vitro* results: superoxide production was not increased as the redox potential of the heme was decreased.

We have yet to find any evidence that changes to the physical properties of the heme can lead to an increased production of superoxide, either *in vivo* or *in vitro*. At this point, it is highly unlikely that the heme is actively involved in ROS suppression in this enzyme.

### EPR Characterization of Heme *b* in Variant Sdh Enzymes

**Figure 2:**
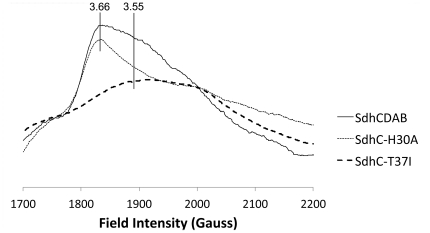
Ferric heme EPR lineshapes of the two heme conformations. The signal in wild-type Sdh is a mixture of a sharp and broad species, exemplified in the SdhC^H30A^ and SdhC^T37I^ variants, respectively.

The *b* heme of *E. coli* Sdh exhibits a highly anisotropic low spin (HALS)-type EPR signal characterized by a large g_z_ feature (g_z_>3) [23]. The HALS signal is a result of strain on the heme iron, due to the near perpendicular orientation of the imidazole sidechains belonging to the two histidine ligands [24], [25]. In wild-type Sdh, the EPR lineshape of the ferric heme consists of two components that likely arise from two distinct conformations of the heme. As shown in **Figure 2**, the heterogeneous signal observed is a combination of a sharp signal at g_z_ = 3.66 and a broad signal centered at g_z_ = 3.55. Mutations such as SdhC^H30A^ can shift the equilibrium towards the sharp species, whereas the EPR spectrum of the SdhC^T37I^ mutation is almost entirely the upfield broad species. Due to the high g anisotropy of these HALS-type hemes, g_x_ and g_y_ values are difficult to measure.

**Figure 3:**
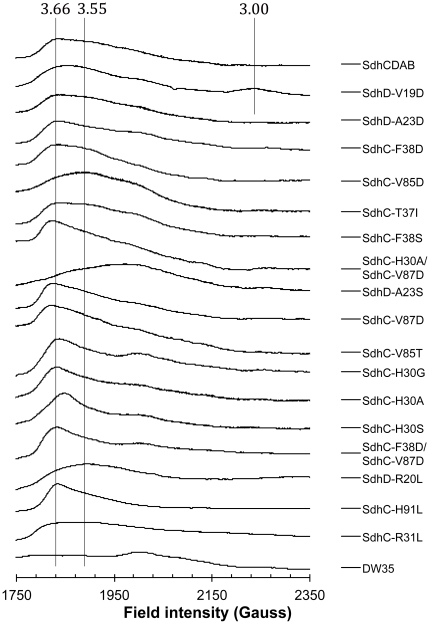
EPR Spectra of low-spin heme. Crude membranes enriched in wild-type SdhCDAB and Sdh variants were oxidized with 0.8 mM DCPIP. DW35 membranes do not contain Sdh enzyme and serve as the negative control. Spectra were recorded at 20 mW at 12 K.

**Figure 3** shows the EPR spectra of the oxidized heme in all the Sdh variants included in this study. The SdhD^V19D^ mutation resulted in the most dramatic change in the EPR lineshape of the heme, but this effect was unique to this enzyme. Here, we observed the appearance of a new rhombic species at g = 3.00 in addition to the regular signal in the g = 3.5–3.6 range and these two signals both titrate to the same midpoint potential (**Table 1**). Given the low g anisotropy of this new species, this signal likely arises from an isomeric fraction of the enzyme in which the two histidine ligands are coplanar. The addition of an Asp residue in position 19 of SdhD likely disrupts the normal orientation of the nearby SdhD^H71^ ligand during assembly of the heme, leading to the incorporation of other rotamers of that imidazole sidechain.

In these experiments and previous studies by Cheng et al. [12], we observed Sdh variants that appear to shift the equilibrium towards one of the two conformational states that exist in wild-type Sdh (**Figure 3**). The positions of these altered amino acids and their consequences on the EPR signals provide indirect evidence that allow us to speculate that the different EPR signals are a consequence of out-of-plane distortions of the porphyrin ring. Although energetically unfavorable, distortion of the heme away from planarity is common in Fe-porphyrins [26] and is heavily influenced by the surrounding protein milieu [27], [28].

Out-of-plane distortions of the porphyrin ring are normally dependent on hydrogen bonding with the heme propionates and van der Waals contacts with the porphyrin ring. In model heme systems, possible distortions include saddled, ruffled, domed, waved, and propellering conformations [29], [30], although it is the two former configurations that are most commonly observed. We believe that a saddled porphyrin ring is the origin of one of the observed EPR lineshapes, while the other is due to a more planar orientation of the porphyrin. This is consistent with more recent crystal structures of the *E. coli* Sdh, which modeled the heme in a more saddled conformation [31]. This is in contrast to the initial 1NEK structure that modeled the heme in a near planar conformation [4]. While the high resolution structural data hints at the two conformational states, the following biophysical data also suggests that this phenomenon is real and not an artefact of the crystallography.

**Figure 4:**
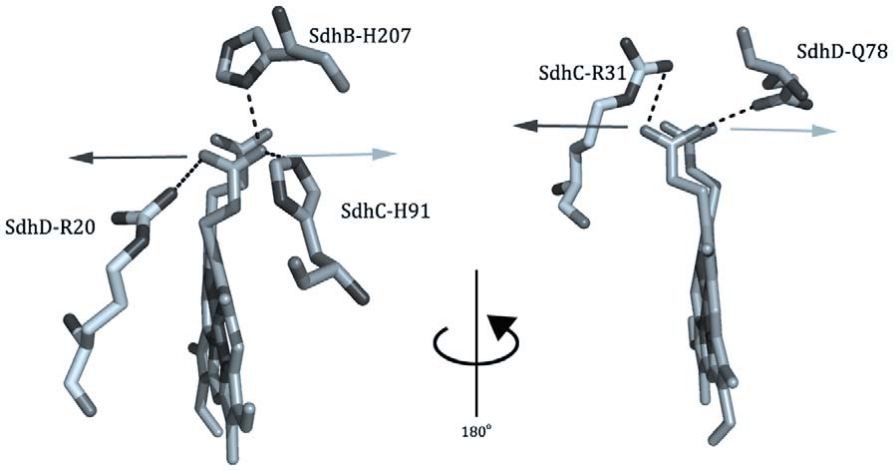
The hydrogen-bonding network around the heme propionates. The distal propionate is highlighted on the left while the proximal propionate is shown on the right. Each propionate is held in position by residues on either side. Mutation of SdhD^R20^ and SdhC^R31^ to a Leu results in the propionate shifting towards the other “girder” (light arrows). Mutation of SdhC^H91^ and SdhD^Q78^ to a Leu causes a shift of the propionate in the other direction (dark arrows). Note the twisting of the porphyrin ring into a saddled conformation in this structure. (pdb: 2WDV [31]).

An examination of the hydrogen-bonding network around the heme propionates reveals some interesting results. SdhC^H91^, SdhB^H207^, and SdhD^R20^ form a hydrogen-bonding network with the propionate distal to the Q-site. SdhD^Q78^ and SdhC^R31^ are hydrogen-bonded to the proximal propionate (**Figure 4**). In the 2WDV X-ray structure containing the saddled heme, the pyrrole ring associated with the distal propionate is shifted towards SdhD^R20^ while the proximal pyrrole ring contorts in the opposite direction, towards SdhD^R31^. Along the pseudo two-fold axis of the heme, SdhD^R20^ and SdhC^R31^ are in equivalent positions, as are SdhC^H91^ and SdhD^Q78^. These residues surrounding the heme propionates are generally well conserved among different complex II homologs.

Mutations around the heme propionates reveal an interesting symmetry. The SdhD^R20L^ and SdhC^R31L^ mutations result in a shift towards the broad EPR spectrum at g_z_ = 3.55 (*E_m,7_* = −25 mV and +15 mV, respectively), although the effect is not as pronounced in the SdhD^R20L^ variant. Conversely, the SdhC^H91L^ and SdhD^Q78L^ [12] mutations (*E_m,7_* = −30 mV and +8 mV [12], respectively) both cause a shift towards the sharp species at g_z_ = 3.66 (**Figure 3**). The mutation of any one of these “girder” residues is likely to cause the heme propionate to shift towards the remaining residue, resulting in the equilibrium shift towards planarity or distortion. For example, in the case of the SdhC^H91L^ mutation, the distal propionate will shift towards SdhD^R20^, and vice versa. When considering mutations to the hydrogen-bonding network of the propionates, the symmetry strongly suggests that one of the species seen by EPR is the heme in a saddled conformation, as this particular distortion is characterized by the same two-fold symmetry. The crystal structure of the SdhB^H207T^ was recently elucidated [32] and shows the distal propionate shifted towards SdhD^R20^. This is the same configuration that is expected in the SdhC^H91L^ mutation and consistently, both the SdhC^H91L^ and SdhB^H207T^ variants (*E_m,7_* = +20 mV [32]) exhibit the same sharp EPR spectra. Additionally, in the SdhB^H207T^ variant, the heme in that crystal structure has been modeled as less distorted than in the 2WDV structure, which shows the most heme distortion. This suggests that the sharp EPR species may represent a relatively more planar conformation.

**Figure 5:**
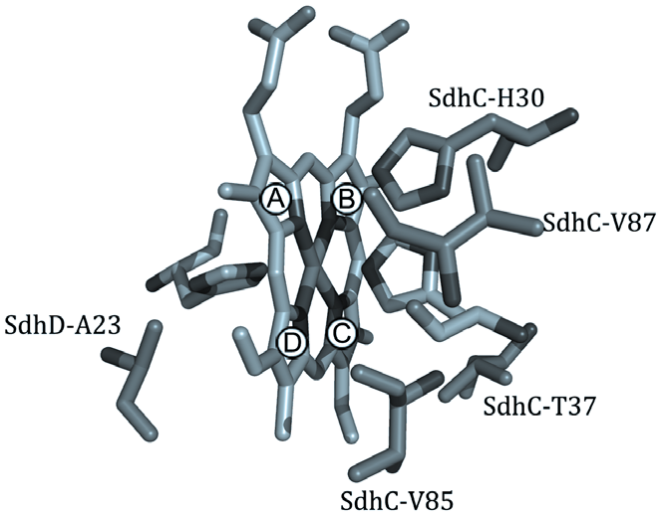
Interactions between the heme face and surrounding residues. Mutations to the residues depicted are able to affect the planarity of the porphyrin ring. Pyrrole rings are denoted as (A) top, distal (B) top, proximal (C) bottom, proximal (D) bottom, distal. (pdb: 2WDV [31]).

Mutations to amino acids packed against the face of the porphyrin ring can also shift the equilibrium between the saddled and planar conformations. SdhC^H30^ is in close van der Waals distance (∼3.5 Å) to the top, distal pyrrole ring of the heme (ring A, **Figure 5**) (we refer to the propionate end as the top of the heme). In all the crystal structures, this pyrrole ring appears to be most distorted from planarity, and the position of SdhC^H30^ suggests that this residue strongly contributes to the out-of-plane distortion of that ring. We expect the loss of this residue will relieve steric forces on the heme and result in a more planar orientation of the porphyrin molecule. Consistent with this, when we mutated SdhC^H30^ we noted a shift in equilibrium towards the sharp EPR species.

The SdhC^V87D^ mutation also results in the observation of the sharp species. This residue is located at a farther distance from the heme (9 Å) but the Asp residue here likely interacts with the nearby SdhC^H30^ residue, pulling the latter residue away from the heme, and leading to increased planarity in the porphyrin ring. Considering these results and the SdhB^H207T^ crystallographic data, we can confidently assign the sharp EPR species as originating from a more planar conformation of the heme, whereas the broad component of the lineshape is derived from a more saddled distortion.

Interestingly, mutations along the bottom edge of the heme are also able to elicit the same spectroscopic shifts as those along the top edge of the heme. Here, the SdhD^A23S^ mutation gives rise to a broad signal, however, the g_z_ value is shifted upfield to g = 3.40. This shift is likely a result of interactions between the serine hydroxyl and the nearby heme ligand, SdhD^H71^, which may reduce the perpendicularity between the two histidine ligands. SdhC^V85^ lies at an equidistant position on the opposing side of the bottom, distal pyrrole ring (ring D, **Figure 5**) as SdhD^A23^ and we can observe a similar symmetry in the EPR spectra as with the mutations around the heme propionates. In a saddled conformation, the distal, bottom pyrrole ring would be displaced away from planarity towards Sdh^CV85^. The introduction of a polar Ser or Thr juxtaposed with the relatively hydrophobic pyrrole ring should displace the ring away from the hydroxyl. Thus, the SdhC^V85T^ mutation should increase planarity (sharp EPR species) while the SdhD^A23S^ mutation should decrease planarity (broad EPR species). Our results are consistent with this. Unexpectedly, when an Asp residue is mutated into either of the two positions, we did not see a change in the EPR spectroscopic characteristics of the heme. Here, is it likely that the increased length of the Asp sidechain positions the carboxyl too far away to interact with the neighboring pyrrole ring.

SdhC^T37^ is situated next to the proximal, bottom pyrrole ring (ring C, **Figure 5**), but its hydroxyl group is pointed away from the heme and as such the dipole here may not be interacting directly with the porphyrin ring. The SdhC^T37I^ mutation introduces a much bulkier sidechain at this position, which would force the proximal, bottom pyrrole ring away for steric reasons, favoring a more saddled porphyrin conformation. Hence, we observe the broad EPR species in this variant (g_z_ = 3.55).

Of note, mutations associated with increased planarity of the porphyrin molecule are also associated with lower midpoint potentials. Distorted porphyrin systems result in decreased electron density at the heme iron and an increase in redox potential [33]. This trend does not hold when considering mutations around the propionates but this is likely due to unpredictable changes in the hydrogen-bonding network. However, there is consistent support for saddled distortions in the *E. coli* Sdh heme. Future crystallographic studies on these Sdh variants will be needed to provide direct evidence of heme distortion.

### Summary

We made mutations in *E. coli* Sdh that modulated the midpoint potential of the heme by nearly 100 mV and assessed the effect on the catalytic ability of the enzyme. Although many of our variants had subtle effects on activity compared to wild-type Sdh, we could not observe a correlation between heme *E*_m_ and catalytic turnover. In addition, we did not find evidence that the heme is involved in the suppression of ROS, either *in vitro* or *in vivo*.

Studies have shown that in the c-type cytochromes, out-of-plane distortions can be conserved among protein homologs across different organisms [30]. The conservation of what is an energetically unfavorable process suggests some purpose for such higher order structures. Here, an examination of the biophysical data suggests that in the ferric state, the heme cofactor in wild-type Sdh exists in a heterogenous population of saddled and planar conformations. The saddling of the heme likely contributes to its high midpoint potential in this particular complex II homolog.

In this study, we did not observe a link between heme structure and *in vitro* turnover, but since the heme can adopt at least two physically distinct forms, it is an enticing possibility that redox-linked conformational changes may be a possible mechanism to regulate some aspect of enzyme activity, *in vivo*. Heme conformations can be affected by redox state due to the differences in pyrrole (Fe-N) bond lengths between the ferric and ferrous states [34], [35]. Already, it has been demonstrated that other cytochromes are capable of large-scale conformational changes associated with the redox state of their heme cofactors [36], [37]. A conformational shift between a saddled and planar state during redox cycling could be communicated throughout the structure of the membrane domain, given the face of the heme is in van der Waals contact with a large portion of the transmembrane helices. As well, the heme propionates are hydrogen bonded to key residues that constitute the Q-site and minor changes here could impact the enzyme affinity for the ubiquinone pool. If the heme is acting as a molecular sensor of the redox state of the quinone pool *in vivo*, a redox-induced conformational change could provide a possible mechanism.

## Supporting Information

Figure S1 **SDS-PAGE gels of Sdh-enriched membranes.** Expression levels of each Sdh subunit are similar in all the mutants. Lane 1, SdhCDAB. Lane 2, SdhD^V19D^. Lane 3, SdhD^A23D^. Lane 4, SdhC^F38D^. Lane 5, SdhC^V85D^. Lane 6, SdhC^T37I^. Lane 7, SdhC^F38S^. Lane 8, SdhC^H30A^/SdhC^V87D^. Lane 9, SdhD^A23S^. Lane 10, SdhC^V87D^. Lane 11, SdhC^V85T^. Lane 12, SdhC^H30G^. Lane 13, SdhC^H30A^. Lane 14, SdhC^H30S^. SdhC^F38D^/SdhC^V87D^.
(TIF)

Figure S2 **Succinate-reduced minus air-oxidized absorbance spectrum.** The 100 mV decrease in heme *E*_m_ in the SdhC^F38D^/SdhC^V87D^ mutant significantly diminishes its succinate reduction capacity. The DW35 membranes were reduced with dithionite to show levels of *b*-type hemes in cytochromes *bo*_3_- and *bd*- oxidase. Sdh-enriched membranes are expected to have less contaminating *b*-type hemes due to Sdh overexpression.
(TIF)

Table S1 **List of mutagenic oligonucleotides.** A universal primer (M13 – forward or reverse) was used in combination with the appropriate mutagenic oligonucleotide in each PCR reaction.
(DOC)
