# Supplementary figures and images for: Out of Plane Distortions of the Heme b of Escherichia coli Succinate Dehydrogenase
Source: PLoS One. 2012 Feb 29;7(2):e32641. doi: 10.1371/journal.pone.0032641 (PMC3290573; doi:10.1371/journal.pone.0032641)

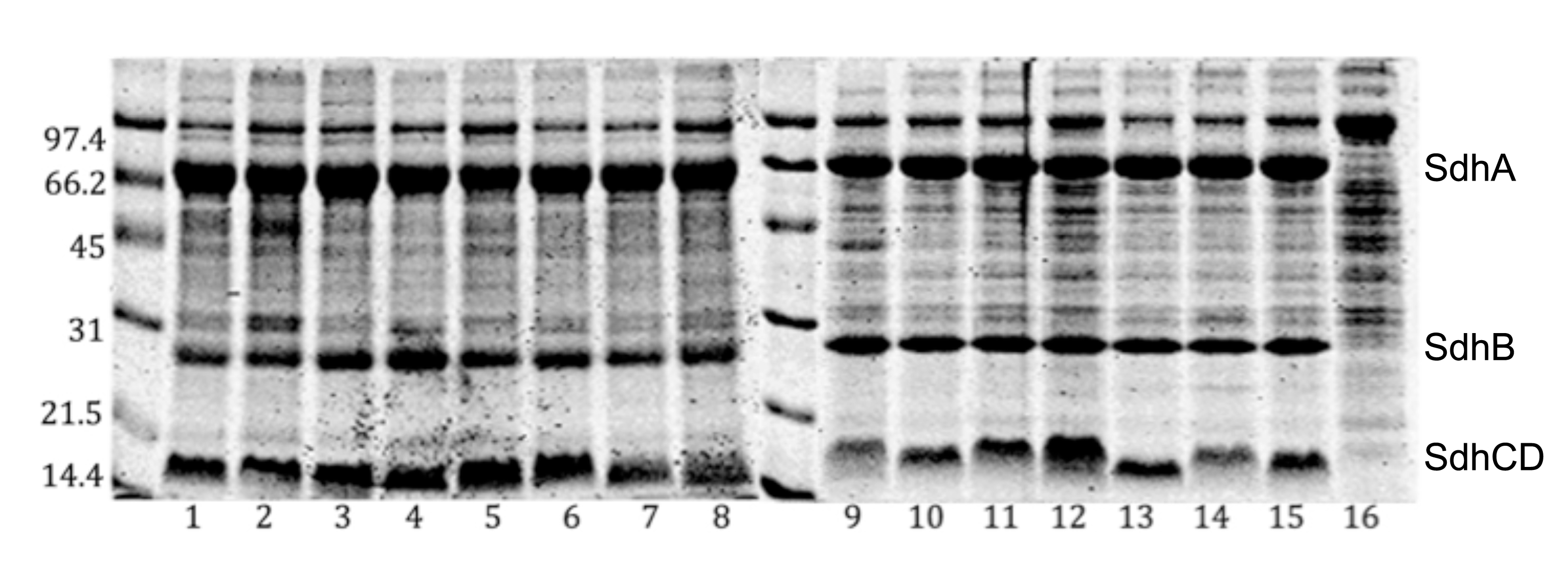

Supplement: Figure S1 — SDS-PAGE gels of Sdh-enriched membranes. Expression levels of each Sdh subunit are similar in all the mutants. Lane 1, SdhCDAB. Lane 2, SdhDV19D. Lane 3, SdhDA23D. Lane 4, SdhCF38D. Lane 5, SdhCV85D. Lane 6, SdhCT37I. Lane 7, SdhCF38S. Lane 8, SdhCH30A/SdhCV87D. Lane 9, SdhDA23S. Lane 10, SdhCV87D. Lane 11, SdhCV85T. Lane 12, SdhCH30G. Lane 13, SdhCH30A. Lane 14, SdhCH30S. SdhCF38D/SdhCV87D. (TIF) [file pone.0032641.s001.tif]

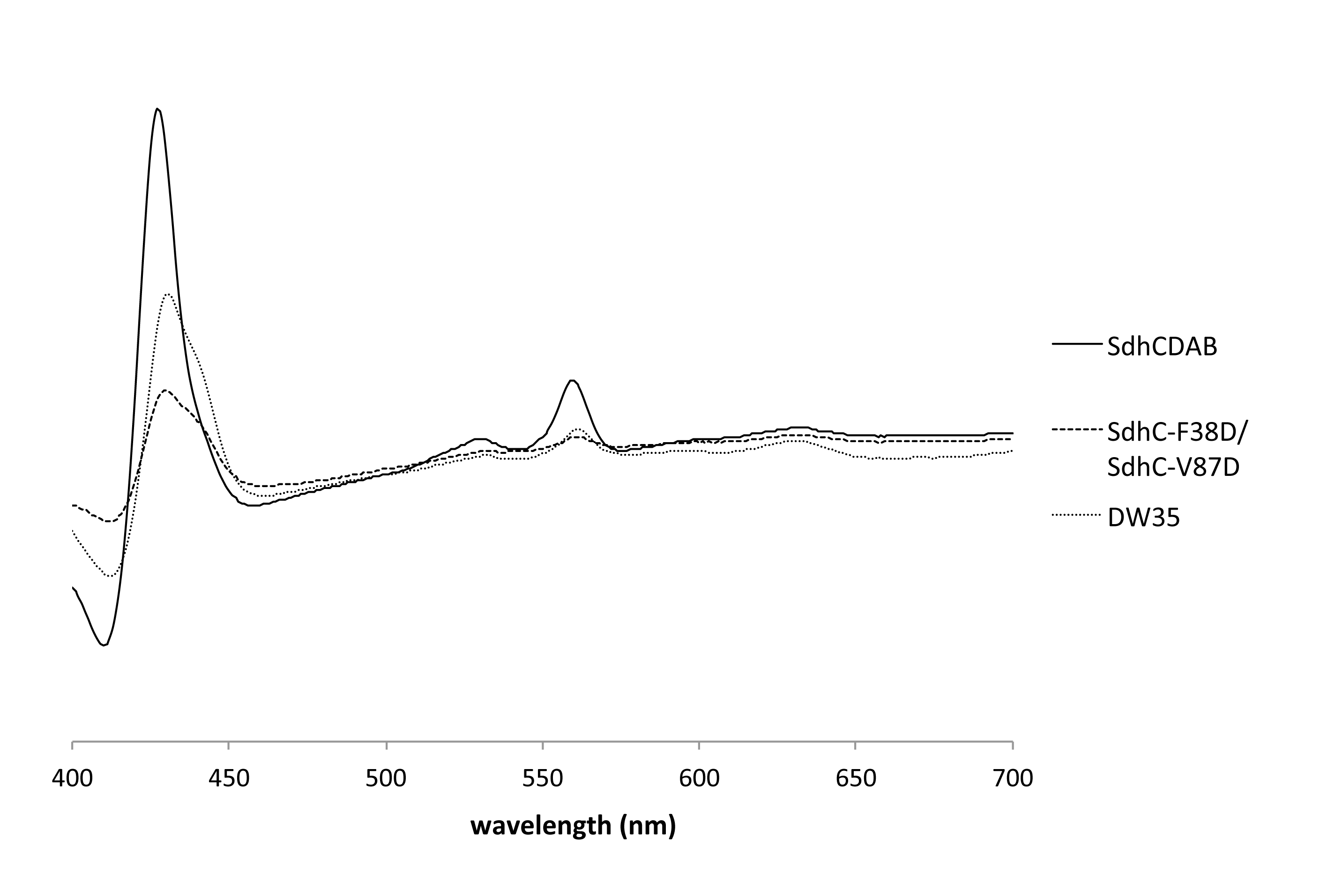

Supplement: Figure S2 — Succinate-reduced minus air-oxidized absorbance spectrum. The 100 mV decrease in heme E m in the SdhCF38D/SdhCV87D mutant significantly diminishes its succinate reduction capacity. The DW35 membranes were reduced with dithionite to show levels of b-type hemes in cytochromes bo 3- and bd- oxidase. Sdh-enriched membranes are expected to have less contaminating b-type hemes due to Sdh overexpression. (TIF) [file pone.0032641.s002.tif]
