# Supplementary material for: Out of Plane Distortions of the Heme b of Escherichia coli Succinate Dehydrogenase
Source: PLoS One. 2012 Feb 29;7(2):e32641. doi: 10.1371/journal.pone.0032641 (PMC3290573; doi:10.1371/journal.pone.0032641)
Supplement: Table S1 — List of mutagenic oligonucleotides. A universal primer (M13 – forward or reverse) was used in combination with the appropriate mutagenic oligonucleotide in each PCR reaction. (DOC) [file pone.0032641.s003.doc]

| Mutant | Mutagenic Oligonucleotide (5' to 3') |
| --- | --- |
| SdhD-V19D | GATTTCATCCTCGATCGCGCTACCGC |
| SdhD-A23D | CGTTCGCGCTACCGATATCGTCCTGACG |
| SdhC-F38D | GTGTGATCACCGATGTTGCAGTGGGGATCCTGC |
| SdhC-V85D | CTGGCGTATCACGACGTCGTAGGTATTCG |
| SdhC-T37I | CCGGTGTGATCATCTTTGTTGCAGTGGGGATCCTGC |
| SdhC-F38S | GTGTGATCACCGATGTTGCAGTGGGGATCCTGC |
| SdhD-A23S | CGTTCGCGCGACGTCTATCGTCCTGACG |
| SdhC-V87D | GTATCACGTCGTCGACGGTATTCGCCAC |
| SdhC-V85T | CTGGCGTATCACACCGTCGTAGGAATTCGCCAC |
| SdhC-H30G | CCGGAAACGCGTCCGAGAATGGACGCTATC |
| SdhC-H30A | CACACCGGAAACGCGTGCGAGAATGGACGC |
| SdhC-H30S | CACACCGGAAACGCGTGAGAGAATGGACGC |
| SdhC-R31L | TCCATTCTCCATCTGGTTTCCGGTGTGATCACC |
| SdhC-H91L | TATTCGCCTCATGATGAT |
| SdhD-R20L | GATTTCATCCTCGTTCTGGCTACCGCTATCG |
